# Synergistic Reversal of Intrahepatic HCV-Specific CD8 T Cell Exhaustion by Combined PD-1/CTLA-4 Blockade

**DOI:** 10.1371/journal.ppat.1000313

**Published:** 2009-02-27

**Authors:** Nobuhiro Nakamoto, Hyosun Cho, Abraham Shaked, Kim Olthoff, Mary E. Valiga, Mary Kaminski, Emma Gostick, David A. Price, Gordon J. Freeman, E. John Wherry, Kyong-Mi Chang

**Affiliations:** 1 Philadelphia Veterans Affairs Medical Center, Philadelphia, Pennsylvania, United States of America; 2 Division of Gastroenterology, Department of Medicine, University of Pennsylvania School of Medicine, Philadelphia, Pennsylvania, United States of America; 3 Department of Surgery, Penn Liver Transplant Center, Hospital of the University of Pennsylvania, Philadelphia, Pennsylvania, United States of America; 4 Department of Medical Biochemistry and Immunology, Cardiff University School of Medicine, Cardiff, United Kingdom; 5 Department of Medical Oncology, Dana Farber Cancer Institute and Department of Medicine, Harvard Medical School, Boston, Massachusetts, United States of America; 6 Immunology Program, The Wistar Institute, Philadelphia, Pennsylvania, United States of America; University of Washington, United States of America

## Abstract

Viral persistence is associated with hierarchical antiviral CD8 T cell exhaustion with increased programmed death-1 (PD-1) expression. In HCV persistence, HCV-specific CD8 T cells from the liver (the site of viral replication) display increased PD-1 expression and a profound functional impairment that is not reversed by PD-1 blockade alone. Here, we report that the inhibitory receptor cytotoxic T lymphocyte-associated antigen-4 (CTLA-4) is preferentially upregulated in PD-1^+^ T cells from the liver but not blood of chronically HCV-infected patients. PD-1/CTLA-4 co-expression in intrahepatic T cells was associated with a profound HCV-specific effector dysfunction that was synergistically reversed by combined PD-1/CTLA-4 blockade *in vitro*, but not by blocking PD-1 or CTLA-4 alone. A similar effect was observed in circulating HCV-specific CD8 T cells with increased PD-1/CTLA-4 co-expression during acute hepatitis C. The functional response to combined blockade was directly associated with CTLA-4 expression, lost with CD28-depletion and CD4-independent (including CD4^+^FoxP3^+^ Tregs). We conclude that PD-1 and CTLA-4 pathways both contribute to virus-specific T cell exhaustion at the site of viral replication by a redundant mechanism that requires combined PD-1/CTLA-4 blockade to reverse. These findings provide new insights into the mechanisms of virus-specific T cell dysfunction, and suggest that the synergistic effect by combined inhibitory receptor blockade might have a therapeutic application against chronic viral infection *in vivo*, provided that it does not induce autoimmunity.

## Author Summary

Hepatitis C virus (HCV) is an important human pathogen with a high rate of persistence associated with chronic liver disease that can progress to cirrhosis and hepatocellular carcinoma. Chronic HCV infection occurs in the setting of impaired antiviral T cells that over-express an inhibitory receptor PD-1 (programmed death-1 receptor). Recent studies showed that *in vitro* inhibition of the PD-1 pathway via an inhibitory antibody can reverse the functional impairment in HCV-specific CD8 T cells from blood but not the liver (the site of viral infection and disease progression). In this study, we show that a second co-inhibitory receptor, CTLA-4, is upregulated in HCV-specific CD8 T cells from the liver and that combined PD-1/CTLA-4 blockade (but not single blockade of PD-1 or CTLA-4) can synergistically enhance their function. This functional enhancement was CD28-dependent but CD4-independent. This effect also differed between viruses, tissue compartments (liver vs. periphery) and clinical status (acute vs. chronic). We conclude that PD-1, CTLA-4, and CD28 expression profiles define a novel hierarchy in HCV-specific CD8 T cell exhaustion than can be synergistically reversed by combined inhibitory receptor blockade. These findings have potential immunotherapeutic applications, provided that no autoimmunity is induced.

## Introduction

Virus-specific CD8 T cells become progressively exhausted during chronic viral infection due to increased level or duration of antigenic stimulation without sufficient CD4 help[1]. Among the CD28 family of costimulatory molecules, programmed death-1 (PD-1) is an immune inhibitory receptor that is highly expressed on both exhausted and activated T cells[2]. Interactions between PD-1 and its ligands PD-L1/PD-L2 can inhibit antigen-specific T cell proliferation and effector function[2],[3]. Importantly, blockade of PD-1 signaling can restore function to exhausted virus-specific CD8 T cells with reduced viral load in mice with chronic lymphocytic choriomeningitis virus (LCMV) infection *in vivo* [4], thereby raising the possibility that immune exhaustion can be reversed with potentially therapeutic antiviral effects. A role for PD-1 pathway in viral persistence and antiviral T cell exhaustion has been shown in various chronic viral infections including hepatitis B virus (HBV), human immunodeficiency virus (HIV), simian immunodeficienty virus (SIV) and hepatitis C virus (HCV)[2],[5],[6],[7],[8].

In particular, HCV is a highly persistent human pathogen that infects the liver and causes significant morbidity and mortality due to chronic liver disease[9]. Patients with chronic HCV infection harbor dysfunctional antiviral T cells with increased PD-1 expression in circulating blood, and PD-1 blockade can restore their antigen-specific effector function *in vitro* [10],[11],[12],[13],[14]. However, HCV-specific CD8 T cells in the liver (the site of HCV infection) display markedly increased PD-1 expression compared to peripheral blood [10],[13],[15] and a profound functional impairment that is refractory to PD-1 blockade alone[13]. Similarly, highly activated circulating HCV-specific CD8 T cells in acute evolving hepatitis C show markedly increased PD-1 expression with a deep functional impairment that is unresponsive to PD-1 blockade. These results suggested the existence of additional inhibitory mechanisms that contribute to virus-specific CD8 T cell exhaustion in HCV-infected patients, particularly in PD-1^high^ cells.

Since intrahepatic PD-1^+^ CD8 T cells also express increased levels of immune inhibitory receptor cytotoxic T lymphocyte-associated antigen-4 (CTLA-4)[13], we asked if CTLA-4 might contribute to virus-specific T cell dysfunction in HCV-infected patients. We show that CTLA-4 is preferentially co-expressed in PD-1^high^ CD8 T cells (particularly HCV-specific CD8 T cells) in peripheral blood during acute hepatitis C and in the liver during chronic HCV infection. PD-1/CTLA-4 co-expression was associated with marked antigen-specific effector T cell dysfunction that was dramatically and synergistically reversed by combined PD-1/CTLA-4 blockade *in vitro*. The response to combined PD-1/CTLA-4 blockade was directly associated with CTLA-4 expression, independent of CD4 T cells including FoxP3^+^ Tregs and dependent on CD28 expression. Collectively, these findings suggest that both CTLA-4 and PD-1 pathways contribute to HCV-specific T cell exhaustion in a distinct manner in HCV infection and that combined inhibition of CTLA-4 and PD-1 pathways may have potential therapeutic application in reversing immune exhaustion.

## Results

### CTLA-4 expression is increased in PD-1^+^ HCV-specific CD8 T cells from the liver during chronic HCV infection and in the blood during acute hepatitis C

**Figure 1:**
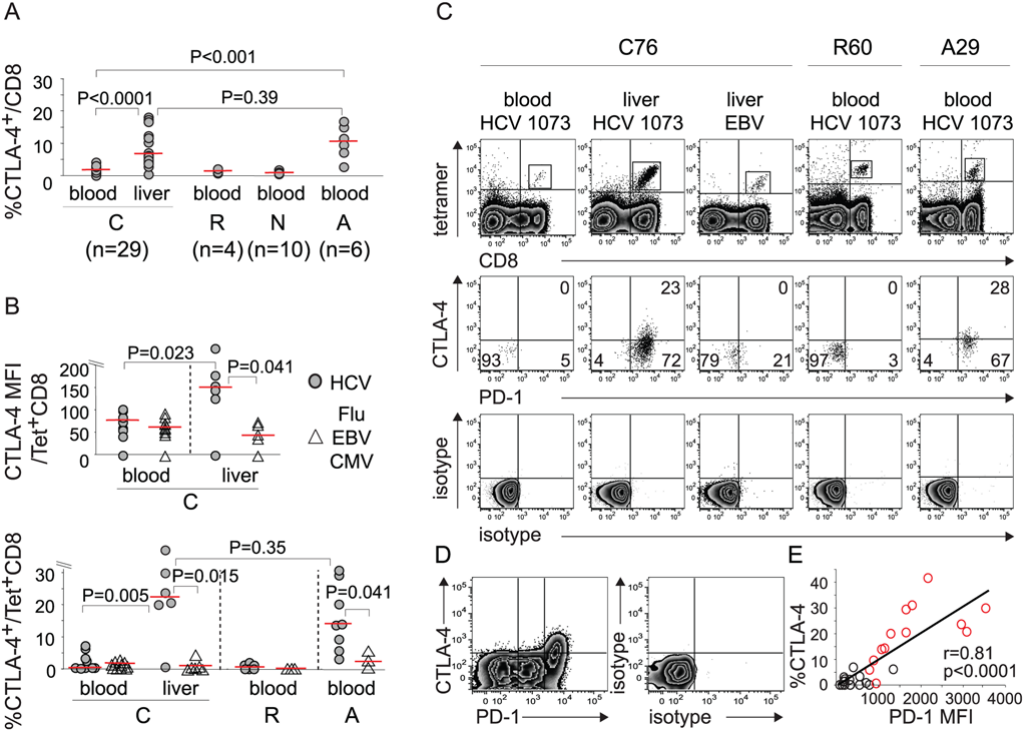
CTLA-4 expression is increased in intrahepatic HCV-specific CD8 T cells. (A) %CTLA-4^+^ in CD8 T cells from 29 chronic (C), 6 acute (A) and 4 resolved (R) hepatitis C patients and 10 HCV-seronegative controls (N). Median: C (blood 1.6%, liver 6.4%); R 1.3%; N 0.9%; A 10.6%. (B) CTLA-4 expression in tetramer^+^ CD8 T cells specific for HLA-A2-restricted HCV (NS3 1073, NS3 1406, NS5 2594) and non-HCV (Flu, CMV and EBV) epitopes from 11C, 3R and 4A patients. Median CTLA-4 MFI: C (blood: HCV 77, non-HCV 61; liver: HCV 151, non-HCV 43). Median %CTLA-4^+^: C (blood: HCV 1.4%, non-HCV 0.9%; liver: HCV 22.2%, non-HCV 0.6%); R (HCV 0.6%, non-HCV 0%); A (HCV 14.0%, non-HCV 2.9%). Red horizontal bars indicate median value. P-values were determined by Mann-Whitney U test. (C) Representative flow cytometry plots. *(Top):* Staining characteristics of tetramer^+^ CD8 T cells. *(Middle):* PD-1/CTLA-4 staining of gated tetramer^+^ CD8 T cells (dot plots). *(Bottom):* PD-1 and CTLA-4 cutoff strategy based on the isotype. (D) Representative FACS plots showing preferential CTLA-4 expression in PD-1-high cells (left) and cutoff strategy based on the isotype (left) in intrahepatic CD8-gated T cells demonstrated with PE-conjugated αPD-1 mAb. (E) Correlation between PD-1 and CTLA-4 expressions in HCV-specific tetramer^+^ CD8 T cells *ex vivo* from HCV-seropositive subjects. Red circles: HCV-specific CD8 T cells from HCV-infected liver and peripheral blood of acute HCV patients.

In chronically HCV-infected patients (C), CTLA-4 expression was greater in CD8 T cells from liver infiltrating lymphocytes (LIL) compared to peripheral blood lymphocytes (PBL) (p<0.0001) (**Figure 1A**). This compartmental difference was further amplified in HCV-specific CD8 T cells both in MFI (p = 0.023) and percentages (p = 0.005), but not in CD8 T cells specific for influenza virus (Flu), cytomegalovirus (CMV) or Epstein-Barr virus (EBV) epitopes (p>0.3) (**Figure 1B**). These expression patterns mirrored PD-1 expression, which is also upregulated in intrahepatic HCV-specific CD8 T cells[10],[12],[13],[16]. Of note, CTLA-4 expression was low in CD8 T cells from liver explants of two HCV-seronegative patients with nonalcoholic steatohepatitis and alcoholic liver disease (**Figure S1**). In peripheral blood, CTLA-4 expression levels were uniformly low in total, HCV-specific and non-HCV-specific CD8 T cells during chronic or resolved (R) HCV infection; this contrasts with PD-1 expression, which is elevated in circulating HCV-specific CD8 T cells from chronic compared to resolved patients[13]. CTLA-4 expression was also upregulated in total and HCV-specific (but not non-HCV-specific) CD8 T cells from the peripheral blood of patients with acute hepatitis C (**Figure 1A/B**). **Figure 1C** shows the representative PD-1 and CTLA-4 expression for peripheral blood and intrahepatic CD8 T cells specific for HCV and non-HCV epitopes detected by HLA-A2/peptide tetramers. Of note, CTLA-4 was preferentially expressed in PD-1-high, but not PD-1-intermediate or PD-1-negative CD8 (**Figure 1D**) and CD4 T cells (data not shown) with a strong association between PD-1 MFI and CTLA-4 expression in percentage (R = 0.81, p<0.0001) (**Figure 1E**) as well as MFI (R = 0.69, p<0.0001, data not shown). Taken together, CTLA-4 expression is induced early with PD-1 in HCV-specific CD8 T cells during acute hepatitis C but becomes compartmentalized to the liver with chronic infection.

### CTLA-4^+^PD-1^+^ CD8 T cells from HCV-infected liver display markedly increased CD28 expression but not ICOS or B and T lymphocyte attenuator (BTLA)

**Figure 2:**
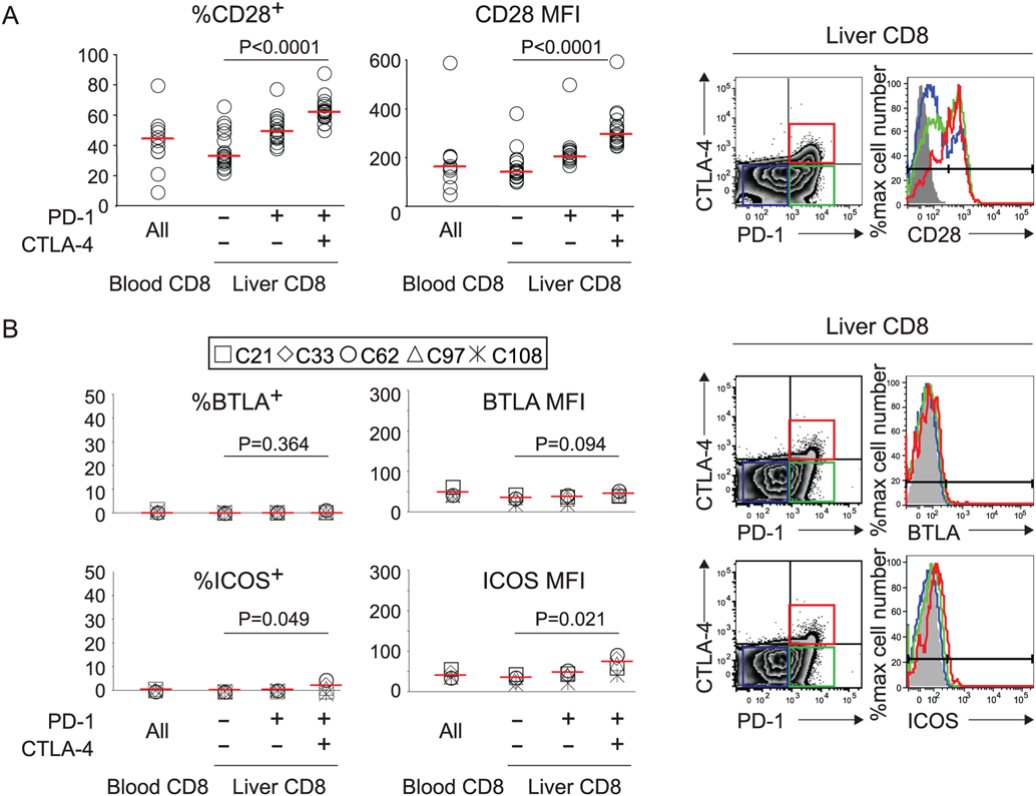
PD-1^+^CTLA-4^+^ CD8 T cells from HCV-infected liver highly express CD28 but not ICOS or BTLA. (A) CD28 expression by percentage and MFI in total CD8 T cells from blood and in liver-derived CD8 T cell subsets (PD-1^−^CTLA-4^−^, PD-1^+^CTLA-4^−^, PD-1^+^CTLA-4^+^) *ex vivo* from 18 chronic HCV patients. Median values (red horizontal lines): %CD28^+^ in intrahepatic PD-1^−^CTLA-4^−^ vs. PD-1^+^CTLA-4^−^ vs. PD-1^+^CTLA-4^+^ subsets (33% vs. 49% vs. 62%, p<0.0001 by the Kruskal Wallis test). (B) BTLA and ICOS expressions by percentage and MFI in total CD8 T cells from blood and in liver-derived CD8 T cell subsets (PD-1^−^CTLA-4^−^, PD-1^+^CTLA-4^−^, PD-1^+^CTLA-4^+^) *ex vivo* from 5 chronic HCV patients. Median values (red horizontal lines): %BTLA^+^ (intrahepatic CD8 subsets: 0.1% vs. 0.4% vs. 0.3%, p = 0.364); %ICOS^+^ (intrahepatic CD8 subsets: 0.1% vs. 0.5% vs. 2.4%, p = 0.049); ICOS MFI (intrahepatic CD8 subsets: 43 vs. 50 vs. 78, p = 0.021). The p-values were calculated by the Kruskal Wallis test. Flow cytometry plots on the right show the characteristic PD-1 and CTLA-4 expression in intrahepatic CD8 T cells (left) and the relative CD28, BTLA and ICOS expression in PD-1^+^CTLA-4^+^ (Red), PD-1^+^CTLA-4^−^ (Green) and PD-1^−^CTLA-4^−^ (Blue) CD8 T cell subsets relative to isotype control (gray shade) in the histograms on the right.

Intrahepatic CD8 T cells were further examined for expression levels of additional CD28 family receptors. As shown (**Figure 2A**), CD28 was highly expressed in PD-1^+^CTLA-4^+^ subset, compared to PD-1^+^CTLA-4^−^ or PD-1^−^CTLA-4^−^ subsets (median 62% vs 49% vs 33%, p<0.0001). By contrast, ICOS and BTLA expression levels were generally low, although a slight increase in ICOS expression was observed in PD-1^+^CTLA-4^+^ subset compared to others (median 2.4% vs 0.5% vs 0.1%, p = 0.049). Thus, intrahepatic CD8 T cells may be subject to inhibitory signals from PD-1 and CTLA-4 as well as a positive signal from CD28 with little contribution from ICOS or BTLA.

### Intrahepatic CD4 T cells display increased CTLA-4 expression without increased FoxP3 expression

CTLA-4 expression was also increased in CD4 T cells from the liver compared to peripheral blood (p<0.0001) (**Figure S3A**). Since CD4^+^FoxP3^+^ Tregs are upregulated in HCV-infected patients and they co-express CTLA-4[17], we asked whether FoxP3^+^ Tregs accounted for increased CTLA-4 expression in intrahepatic CD4 T cells. However, FoxP3^+^ Treg frequencies did not differ between the liver and blood compartments (p = 0.209) (**Figure S3B/C**); furthermore, FoxP3^−^CTLA-4^+^ CD4 T cells were enriched by 2–3 fold in the liver compared to blood (**Figure S3D**). Thus, intrahepatic CD4 and CD8 T cells in HCV-infected patients display increased CTLA-4 expression without increased FoxP3^+^ Treg frequency.

### Combined PD-1/CTLA-4 blockade can synergistically enhance intrahepatic HCV-specific CD8 and CD4 T cell cytokine response

**Figure 3:**
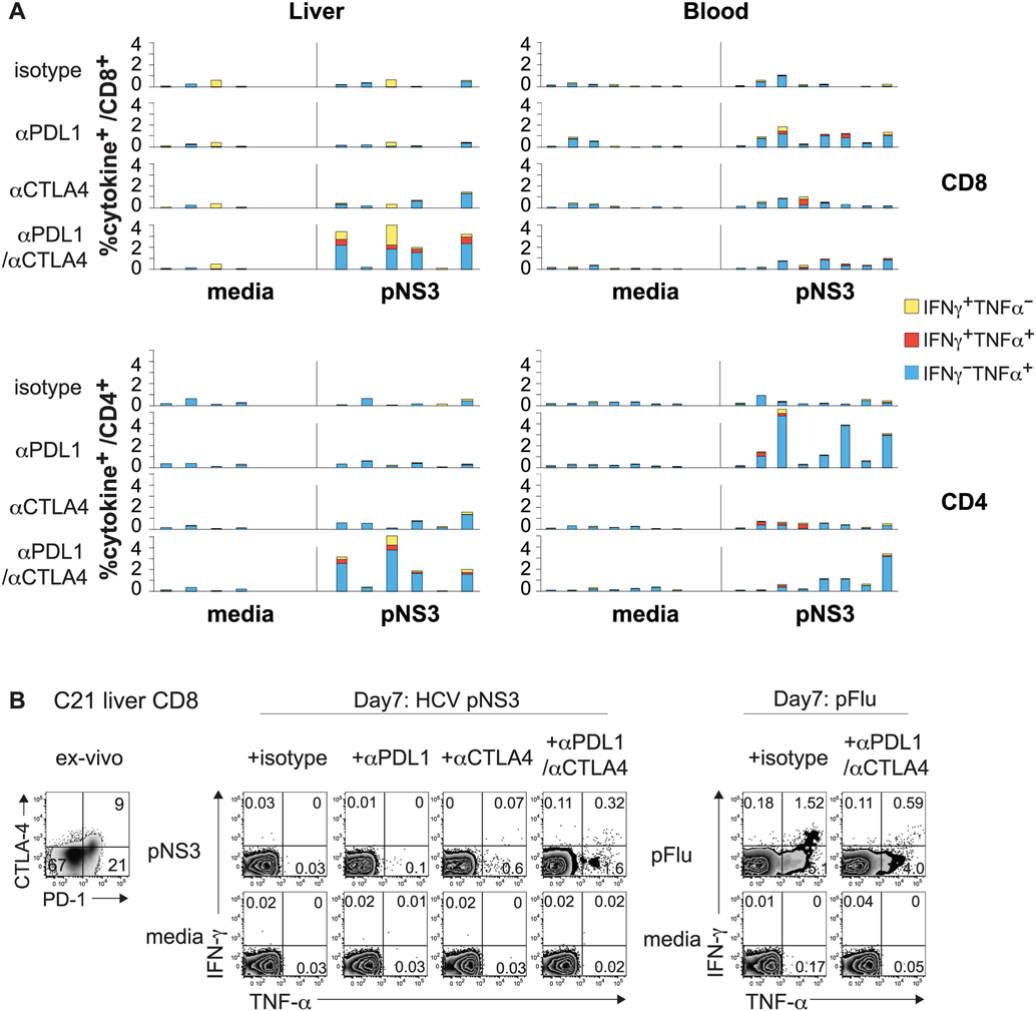
Intrahepatic HCV-specific T cell dysfunction can be reversed synergistically by combined PD-1/CTLA-4 blockade. (A) Effect of inhibitory receptor blockade on HCV-specific IFN-γ and TNF-α production by CD8 and CD4 T cells from liver and blood. The bar graphs show the frequency of CD8 (upper graphs) and CD4 (lower graphs) T cells with HCV-specific intracellular IFN-γ and/or TNF-α expression in liver-derived (left panel, n = 6) and blood-derived (right panel, n = 8) lymphocytes isolated from chronic HCV patients and cultured for 7 days *in vitro* with 15mer overlapping peptides spanning the entire HCV NS3 protein (pNS3) in the presence of isotype control or blocking antibodies. The 7-day cultures were further stimulated for 6 hours with media alone or with pNS3 before intracellular IFN-γ and TNF-α staining. The %IFN-γ^−^TNF-α^+^ (blue bar), %IFN-γ^+^TNF-α^+^ (red bar) and %IFN-γ^+^TNF-α^−^ (yellow bar) T cells are stacked together in each case to show total cytokine^+^ cells. (B) Representative flow cytometry plots showing HCV NS3 and Flu-specific IFN-γ and TNF-α production *in vitro* in liver-derived CD8 T cells from chronic HCV patient C21 following 7 days of culture with NS3 or Flu-derived peptides in the presence of isotype or blocking antibodies. Flow cytometry plots on the far left shows the PD-1 and CTLA-4 expression in liver-derived CD8 T cells directly *ex vivo*.

We previously showed that PD-1 blockade failed to restore function to highly PD-1^+^ HCV-specific CD8 T cells from HCV-infected liver, although it enhanced the functionality of PD-1 intermediate CD8 T cell from blood[13]. As CTLA-4 was preferentially expressed in PD-1^high^ CD8 T cells (**Figure 1D**), we asked if CTLA-4 blockade might reverse their dysfunction, either alone or combined with αPD-L1. To this end, intrahepatic and peripheral lymphocytes from HCV-infected patients were cultured with 15mer peptides spanning the entire HCV NS3 or Flu matrix protein in the presence of blocking αPD-L1 and/or αCTLA-4 or isotype control antibodies. On day 7, the cultures were examined for antigen-specific IFN-γ and TNF-α expression by intracellular cytokine staining. As shown in **Figure 3A**, there was little to no HCV-specific CD4 or CD8 T cell cytokine expression in intrahepatic lymphocytes cultured with αPD-L1 or isotype control antibodies, although low level responses were occasionally seen with αCTLA-4 alone. Remarkably, combined blockade with αPD-L1 and αCTLA-4 resulted in a marked enhancement of intrahepatic HCV NS3-specific CD8 and CD4 T cell cytokine production from 4/6 patients. The comparison of total NS3-specific cytokine responses showed a significant difference between combined PD-1/CTLA-4 blockade and single PD-1 blockade (2.3% vs 0%, p = 0.0037 by Mann Whitney U). However, in blood, HCV-specific CD8 T cell cytokine response was augmented by αPD-L1 without further enhancement by αCTLA-4. Thus, combined PD-1/CTLA-4 blockade resulted in marked HCV-specific cytokine response in LIL but not PBL in HCV-infected patients: LIL 4/6 patients (67%) vs PBL 0/8 patients (0%), p = 0.015 by Fisher's Exact. The effect of inhibitory receptor blockade is shown in representative flow cytometry plots for intrahepatic CD8 (**Figure 3B**); notably, the intrahepatic Flu-specific cytokine response was readily detectable without blockade and not enhanced by PD-1/CTLA-4 blockade (**Figure 3B**, right panels).

### Combined PD-1/CTLA-4 blockade can synergistically enhance intrahepatic HCV-specific CD8 T cell expansion

**Figure 4:**
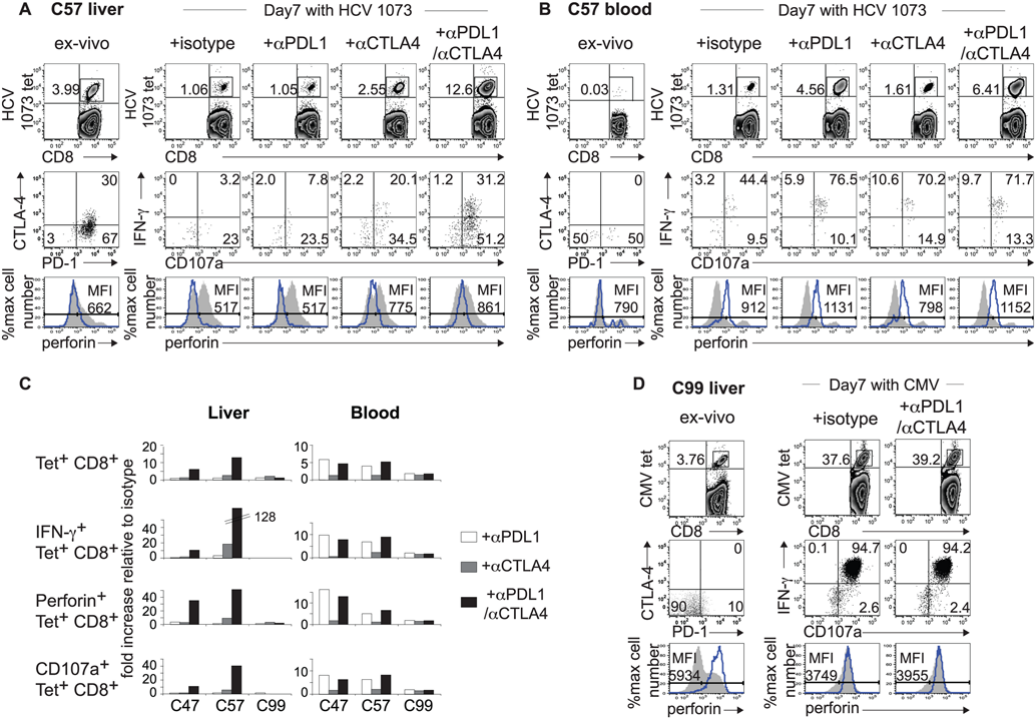
Effect of PD-1/CTLA-4 blockade on intrahepatic and peripheral HCV-specific tetramer+ CD8 T cell function. Flow cytometry plots showing HCV 1073-specific CD8 T cell phenotype directly *ex vivo* and antigen-specific functions following 7 days of antigenic stimulation in the presence of isotype or blocking antibodies, using liver-derived (A) and blood-derived (B) lymphocytes from chronic patient C57. *(Top panels):* frequency of HCV 1073-specific CD8 T cells determined by cognate HLA-A2 tetramer staining. *(Middle panels)* far left: PD-1 and CTLA-4 expression *ex vivo* in gated tetramer^+^ CD8 T cells (dot plots). Remaining right panels: HCV-specific IFN-γ production and CD107a mobilization in gated tetramer^+^ CD8 T cells on day 7. *(Bottom panels):* Perforin expression in tetramer^+^ (blue line) and total CD8 T cells (gray shaded) on day 7. (C) Fold increase in the expansion and effector functions of liver-derived (left) and blood-derived (right) HCV-specific CD8 T cells by αPD-L1 alone (white bar), αCTLA-4 alone (gray bar) and combined αPD-L1/αCTLA-4 blockade (black bar) relative to the isotype control for 3 chronic patients. The frequencies of functional tetramer^+^ CD8 T cells in each culture were calculated by multiplying %tetramer^+^ CD8 T cells with %IFN-γ^+^/tetramer^+^ CD8 T cells, %perforin^+^/tetramer^+^ CD8 T cells or %CD107a^+^/tetramer^+^ CD8 T cells. (D) Flow cytometry plots showing CMV-specific CD8 T cells directly *ex vivo* and their antigen-specific functions following 7 days *in vitro* cultures from chronic patient C99.

The effect of combined PD-1/CTLA-4 blockade on HCV-specific CD8 T cell function was more directly examined in HLA A2+ patients using HLA-A2/peptide tetramers. As shown in **Figure 4A**, αPD-L1 alone did not enhance intrahepatic HCV NS3 1073-specific CD8 T cell expansion compared to isotype in patient C57, and a 2-fold enrichment was observed with αCTLA-4. However, combined PD-1/CTLA-4 blockade induced a dramatic increase in the HCV NS3 1073 tetramer^+^ CD8 T cell frequency compared to isotype control or single blockades with aPD-L1 or aCTLA-4. In peripheral blood, αPD-L1 enhanced HCV-specific CD8 T cell expansion as expected while αCTLA-4 provided only an additive effect (**Figure 4B**). As for antigen-specific effector function, the frequency of tetramer^+^ CD8 T cells with HCV-specific IFN-γ production, CD107a mobilization and perforin expression increased with αCTLA-4 and combined αPD-L1/αCTLA-4 but not with αPD-L1 alone; in peripheral blood, the functional enhancement also occurred with αPD-L1 alone. Overall, the combined PD-1/CTLA-4 blockade enhanced the expansion and effector function of liver-derived HCV tetramer^+^ CD8 T cells compared to isotype control in 2/3 patients (**Figure 4C**). By contrast, intrahepatic CMV-specific CD8 T cells displayed little to no PD-1 or CTLA-4 expression *ex vivo* and were highly functional without further enhancement by PD-1/CTLA-4 blockade (**Figure 4D**). Collectively, these results show that combined PD-1/CTLA-4 blockade can restore proliferative capacity and effector function to deeply exhausted intrahepatic HCV-specific CD8 T cells in a synergistic manner.

### Combined PD-1/CTLA-4 blockade can reverse HCV-specific CD8 T cell dysfunction during acute hepatitis C

**Figure 5:**
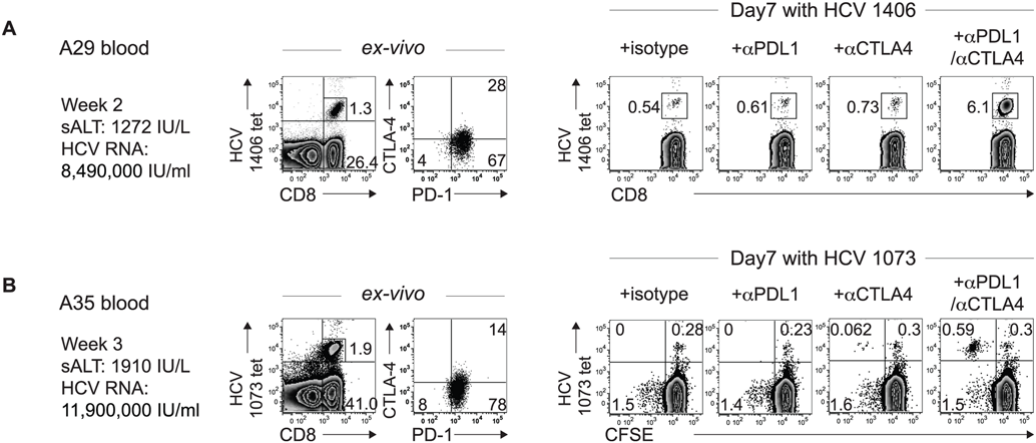
PD-1/CTLA-4 blockade can rejuvenate circulating PD-1^+^CTLA-4^+^ HCV-specific CD8 T cells during acute hepatitis C. *(Left panels):* Flow cytometry plots showing peripheral HCV-specific tetramer^+^ CD8 T cells during acute hepatitis C in patients A29 (A) and A35 (B) *ex vivo*. Gated tetramer^+^ CD8 T cells (dot plots) exhibit increased PD-1 and CTLA-4 expression. *(Right panels):* Flow cytometry plots showing HCV-specific tetramer^+^ CD8 T cell frequency following 7 days of culture with antigenic peptide in the presence of isotype or blocking antibodies for A29. In A35, HCV-specific tetramer^+^ CD8 T cell proliferation was directly monitored in CFSE dilution assay (gating on CD8 T cells) in which the events on the left upper quadrant represent tetramer^+^ CD8 T cells that expanded with CFSE-dilution.

The effect of PD-1/CTLA-4 blockade was examined in 2 HLA-A2+ patients (A29, A35) with acute hepatitis C characterized by markedly elevated serum alanine aminotransferase (sALT) activities and viral titers. As shown (**Figure 5A/B**), circulating HCV-specific CD8 T cells displayed increased PD-1 (A29: 95%; A35: 92%) and CTLA-4 (A29: 28%; A35: 14%) expression. As we previously reported[13], HCV-specific CD8 T cells expanded poorly *in vitro* when stimulated in the presence of αPD-L1 alone. With αCTLA-4 alone, small increases in HCV tetramer^+^ CD8 T cell frequencies were observed in both patients, with proliferation directly measured by CFSE dilution in A35 (**Figure 5B**). However, a marked enhancement of HCV-specific CD8 T cell expansion occurred with combined PD-1/CTLA-4 blockade, mirroring the scenario with intrahepatic T cells. Notably, HCV-specific CD8 T cell dysfunction during acute infection did not persist after spontaneous (A29) or treatment-induced (A35) viral clearance (data not shown), suggesting that PD-1 and CTLA-4 inhibitory pathways can downregulate immune function upon active antigenic encounter, but without necessarily defining the ultimate virological outcome.

### The functional response to combined PD-1/CTLA-4 blockade is associated with CTLA-4 expression on HCV-specific effector CD8 T cells but not FoxP3^+^ Tregs

**Figure 6:**
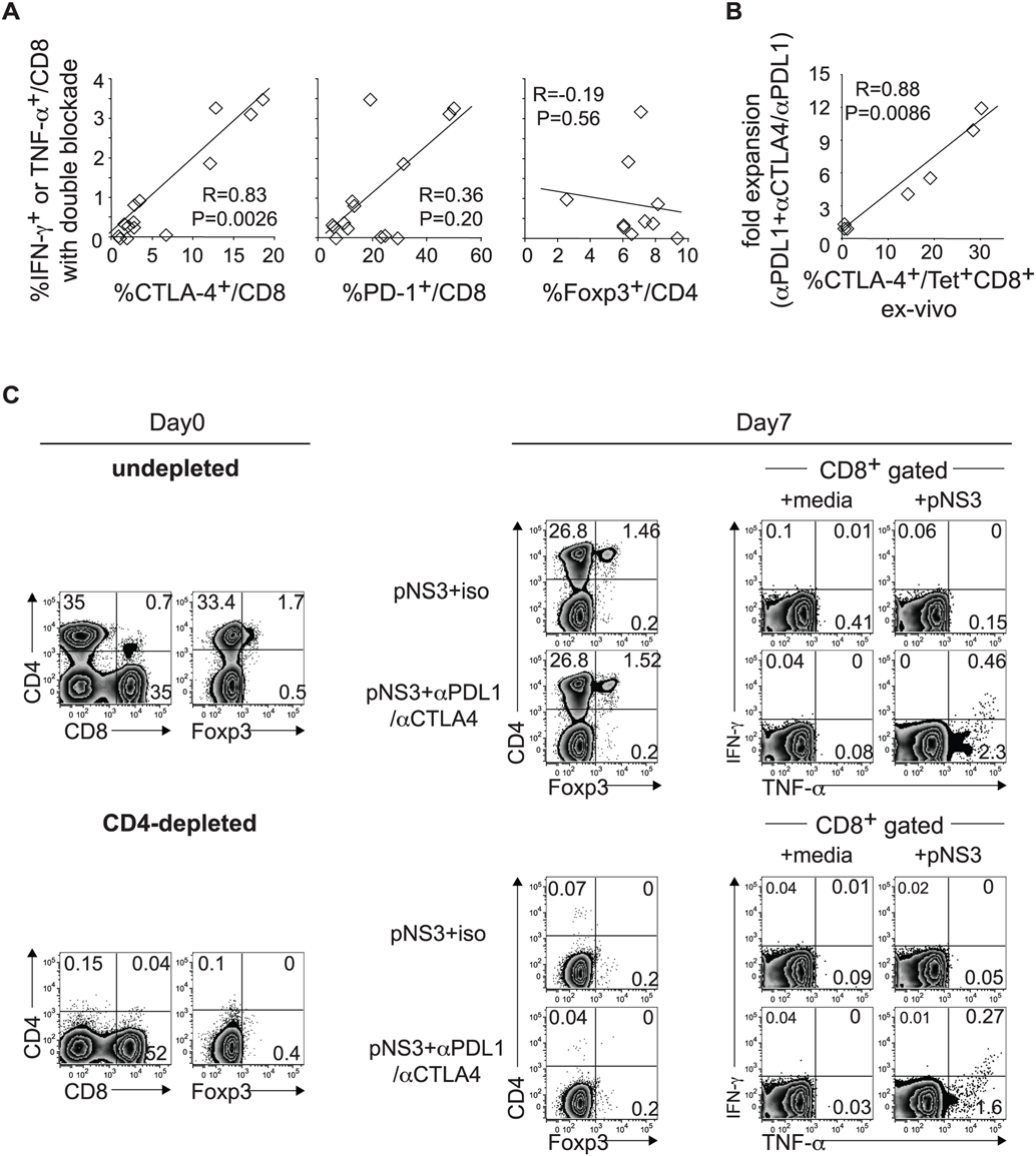
The functional response to PD-1/CTLA-4 blockade associate directly with CTLA-4 expression but not FoxP3^+^ Tregs. (A) Correlation between HCV specific effector cytokine response to combined PD-1/CTLA-4 blockade and *ex vivo* %CTLA-4^+^/CD8 but not %PD-1^+^/CD8 and %FoxP3^+^/CD4. The y-axis represents the sum of CD8 T cells with HCV-specific IFN-γ^+^TNF-α^+^, IFN-γ^+^TNF-α^−^ and IFN-γ^−^TNF-α^+^ expression during combined PD-1/CTLA-4 blockade from 14 HLA-A2^−^ patients (6 intrahepatic and 8 peripheral blood responses). R and p-values by the Spearman rank correlation test. (B) Positive correlation between fold expansion of HCV-specific tetramer^+^ CD8 T cells with combined PD-1/CTLA-4 blockade (relative to PD-1 blockade alone) and *ex vivo* %CTLA-4^+^ in HCV-specific tetramer+ CD8 T cells in 7 HLA A2+ HCV-infected patients. R- and p-values by the Spearman rank correlation test. (C) *(Left)*: Liver infiltrating lymphocytes from chronic patient C07 are examined for CD4, CD8 and FoxP3^+^ T cell subsets before (upper) and after (lower) depletion of CD4 T cells by CD4 Dynabeads (Dynal Inc), resulting in >99% depletion of CD4 T cells including FoxP3^+^ CD4 T cells. *(Right)*: Undepleted and CD4-depleted liver infiltrating lymphocytes were cultured for 7 days with overlapping HCV NS3-derived 15mer peptides (pNS3) in the presence of isotype or blocking antibodies before direct staining for T cell subsets (CD4, FoxP3) and following additional 6 hours of stimulation with media alone (negative control) or pNS3 peptides to examine HCV-specific intracellular IFN-γ and TNF-α expression in CD8 T cells. Combined PD-1/CTLA-4 blockade promoted markedly enhanced HCV-specific cytokine response in undepleted and CD4-depleted cultures regardless of FoxP3^+^ Tregs.

The HCV-specific cytokine response to combined PD-1/CTLA-4 blockade was tightly correlated with CTLA-4 expression in CD8 T cells directly *ex vivo* (R = 0.83, p = 0.0026) (**Figure 6A**); a similar positive trend was noted with PD-1 expression, although this did not reach a statistical significance (R = 0.36, p = 0.20). Notably, the HCV-specific cytokine response during combined PD-1/CTLA-4 blockade did not correlate with the Foxp3^+^ Treg frequency directly *ex vivo*. Moreover, the functional restoration by PD-1/CTLA-4 blockade persisted after CD4-depletion that resulted in complete loss of CD4^+^FoxP3^+^ Tregs (**Figure 6C**); these data indicate that the functional response to inhibitory receptor blockade is independent of CD4^+^FoxP3^+^ Tregs. Among tetramer^+^ CD8 T cells, increased PD-1 expression associated with poor augmentation in antigen-specific expansion with the addition of αPD-L1 (data not shown), as previously reported[13]. However, the addition of αCTLA-4 to αPD-L1 resulted in marked expansion of tetramer^+^ CD8 T cells in direct correlation with CTLA-4 expression *ex vivo* (**Figure 6B**), suggesting that αCTLA-4 acts directly on effector CD8 T cells expressing CTLA-4.

### Functional restoration by combined PD-1/CTLA-4 blockade is CD28-dependent

**Figure 7:**
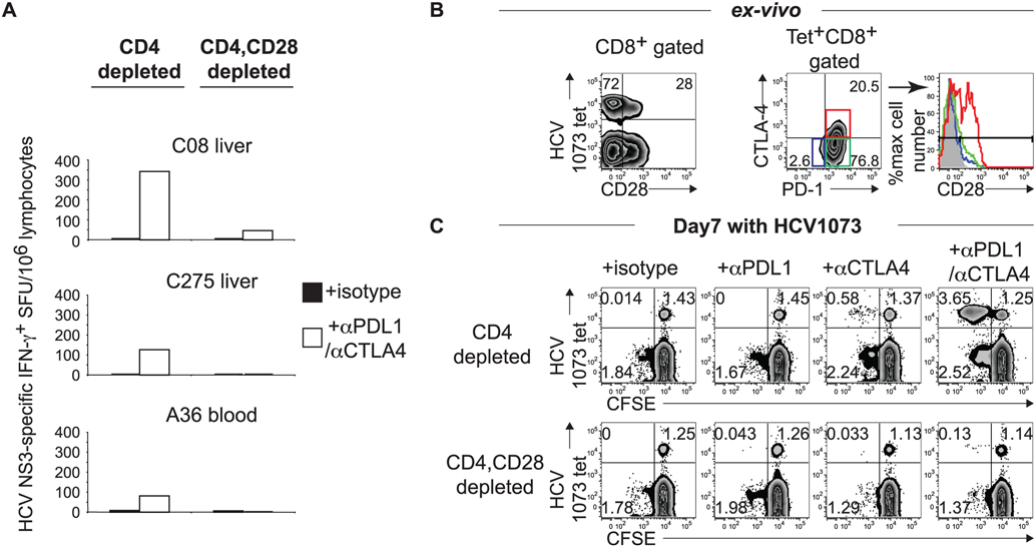
The functional restoration by PD-1/CTLA-4 blockade is CD28-dependent. (A) Loss of HCV-specific CD8 T-cell IFN-γ response by CD28 depletion. LIL or PBL from 3 HLA-A2-negative patients with chronic (C08, C275) and acute (A36) hepatitis C were depleted of CD4 without or with additional CD28 depletion before *in vitro* culture for 7 days with HCV NS3-derived overlapping 15mer peptides in the presence of isotype or PD-1/CTLA-4 blockade. Cultured cells were examined for HCV-specific IFN-γ production in a 45 hour IFN-γ ELISPOT assay. (B) CD28 expression in HCV-specific NS3 1073-specific tetramer^+^ CD8 T-cells relative to PD-1 and CTLA-4 expression *ex vivo*. *(left)* Peripheral HCV 1073-specific tetramer^+^ CD8 T cells from an HLA-A2+ acute HCV patient (A47) display CD28 expression in 28%. *(middle)* Gated HCV 1073-specific tetramer^+^ CD8 T cells show the characteristic PD-1 (97.3%) and CTLA-4 (20.5%) expression. *(right)* Increased CD28 expression in gated PD-1^+^CTLA-4^+^ (Red; 50.0%) HCV tetramer^+^ CD8 T cells compared to PD-1^+^CTLA-4^−^ (Green; 19.5%) and PD-1^−^CTLA-4^−^ (Blue; 12.2%) subsets and isotype control (gray shade) in histogram. (C) Effect of CD28-depletion on antigen-specific expansion in the presence of PD-1 and/or CTLA-4 blockade is shown by CFSE-dilution for HCV NS3 1073-specific tetramer^+^ CD8 T cells from patient A47. CD4 depleted PBL with and without CD28-depletion were CFSE-labeled and stimulated for 7 days in vitro with HCV NS3 1073 peptide in the presence of isotype or blocking antibodies before flow cytometric analysis. Note that HCV tetramer^+^ CD8 T cells remain detectable with CD28 depletion in the bottom graphs.

Since CD28 is over-expressed in PD-1^+^CTLA-4^+^ CD8 T cells (**Figure 2A**) and mediates positive costimulatory signaling for T cell activation [18],[19], we asked if the functional response to PD-1/CTLA-4 blockade is mediated by CD28^+^ T cells. To this end, the effect of PD-1/CTLA-4 blockade on the HCV-specific CD8 T cell IFN-γ response was examined in CD4-depleted lymphocyte subsets with and without CD28-depletion in 3 patients (2 chronic HCV patients with liver-derived lymphocytes, 1 acute HCV patients in peripheral lymphocytes). As shown (**Figure 7A**), the HCV-specific CD8 T cell IFN-γ response was markedly enhanced by combined PD-1/CTLA-4 blockade even in CD4-depleted cells. However, this response was lost with CD28-depletion in all 3 patients, suggesting that the functional response to combined blockade in CD28-dependent.

The role of CD28 in the functional response to PD-1/CTLA-4 blockade was further examined in an HLA-A2+ patient with acute hepatitis C using an HLA-A2/peptide tetramer. As shown in **Figure 7B**, circulating HCV 1073 tetramer+ CD8 T cells in this patient showed 28% CD28 expression in addition to increased PD-1 (97%) and CTLA-4 (21%) expression *ex vivo*. Further subset analysis of HCV 1073 tetramer+ CD8 T cells based on PD-1/CTLA-4 expression showed that CD28 was preferentially expressed in PD-1^+^CTLA-4^+^ (50%) compared to PD-1^+^CTLA-4^−^ (19.5%) or PD-1^−^CTLA-4^−^ (12.2%) subsets (far right histogram on **Figure 7B**), similar to intrahepatic CD8 T cells (**Figure 2A**). As shown in the upper panel (**Figure 7C**), combined PD-1/CTLA-4 blockade markedly enhanced HCV-specific CD8 T cell proliferation in CD4-depleted lymphocytes. With CD28-depletion, this proliferative response was largely lost, even though HCV tetramer+ CD8 T cells remained detectable (**Figure 7C**). Taken together, our results show that both PD-1 and CTLA-4 can co-inhibit HCV-specific CD8 T cell function and that this effect can be reversed by combined PD-1/CTLA-4 blockade. We further show that this functional effect is mediated by CD28^+^ CD8 T cells, independently from CD4^+^ FoxP3+ Tregs.

## Discussion

CTLA-4 is an immune inhibitory receptor within the CD28 family of costimulatory molecules[18],[20],[21]. Induced in activated T cells and constitutively expressed in FoxP3^+^ Tregs[22],[23], CTLA-4 shares its ligands B7-1 and B7-2 with CD28 but binds them with differential kinetics[19],[24],[25]. CTLA-4 inhibits T cell activation by engaging specific signaling pathways and by outcompeting the positive costimulatory receptor CD28[20],[26]. A critical immune regulatory role for CTLA-4 is evident from the massive and fatal lymphoproliferation that occurs in CTLA-4-deficient mice[27],[28]. Antibody-mediated blockade of CTLA-4 signaling can augment antigen-specific CD8 T cell responses in a CD4-independent manner, promoting anti-tumor and autoimmune effects[21],[29]. CTLA-4 also contributes to immune regulation and pathology in animal models of bacterial or parasitic infection[30],[31]. CTLA-4 may play a more variable role in viral infections. For example, in LCMV-infected mice, the disruption of CTLA-4 signaling fails to modify the course of infection or antiviral T cell responses *in vivo* [32],[33], unlike PD-1 blockade[4]. By contrast, increased CTLA-4 expression on HIV-specific CD4 (but not CD8) T cells is strongly associated with disease progression and reversible immune dysfunction in HIV-infected patients[34]. Little is known about the relevance of CTLA-4 in HCV infection. We show here that CTLA-4 (together with PD-1) contributes to HCV-specific T cell dysfunction in HCV-infected liver that can be dramatically reversed by combined CTLA-4 and PD-1 blockade.

There are several notable findings in our study. For example, CTLA-4 was upregulated in deeply exhausted, HCV-specific PD-1^high^ CD8 T cells at the site of viral replication (i.e. liver). The increased CTLA-4 and PD-1 expression was functionally relevant, since intrahepatic HCV-specific CD8 T cells regained their function with combined PD-1/CTLA-4 blockade, although not with single-blockade of PD-1 or CTLA-4. Overall, combined PD-1/CTLA-4 blockade strongly enhanced the HCV-specific CD8 T cell response *in vitro* in 6/9 patients from the liver (4/6 HLA-A2−; 2/3 HLA-A2+), compared to the peripheral blood response which was enhanced in none of the 11 chronically infected patients (p = 0.0016). The combined blockade enhanced antigen-specific T cell cytokine production (e.g. IFN-γ and TNF-α) and cytolytic potential (e.g. perforin expression and CD107a degranulation) as well as their expansion. Thus, PD-1/CTLA-4 blockade promoted a polyfunctional HCV-specific T cell response, perhaps acting beyond the inhibition of cellular apoptosis which is increased in highly PD-1^+^ CD8 T cells in the liver or during acute hepatitis C[35],[36]. Interestingly, while combined blockade enhanced both HCV-specific T cell IFN-γ and TNF-α production (**Figure 3**), the increase was particularly evident for TNF-α, suggesting that PD-1/CTLA-4 blockade may promote a cytokine profile that differs from a preferential (albeit weak) IFN-γ rather than TNF-α production by dysfunctional HCV-specific CD8 T cells in chronic hepatitis C[11]. The combined PD-1/CTLA-4 blockade also enhanced intrahepatic HCV-specific CD4 T cell function, an important consideration given the relevance of CD4 T cells in immune regulation[37],[38].

Although HCV persistence is associated with increased levels of circulating CD4^+^FoxP3^+^ Tregs that constitutively express CTLA-4[17],[39],[40],[41], the level of functional restoration in CD8 T cells by PD-1/CTLA-4 blockade correlated directly with the frequency of CTLA-4^+^ CD8 T cells but not FoxP3^+^CD4^+^ Tregs *ex vivo*. Furthermore, while the functional response to combined blockade differed between the liver and blood, FoxP3^+^ Treg frequencies did not differ between the two compartments *ex vivo* or following *in vitro* culture with PD-1/CTLA-4 blockade (data not shown). Finally, the response to combined blockade persisted after CD4 T cells (including CD4^+^FoxP3^+^ Tregs) were depleted. These results suggest that PD-1/CTLA-4 blockade targets effector T cells directly in a manner independent of CD4 T cells including FoxP3+ Tregs[42].

PD-1 and CTLA-4 inhibit T cell activation through distinct mechanisms that converge on Akt: PD-1 inhibits CD28-mediated activation of phosphatidylinositol 3-kinase (PI3K) and CTLA-4 activates the type II serine/threonine phosphatase PP2A, both leading to the inhibition of Akt phosphorylation[43]. By competing with CD28 for B7-1 and B7-2[24],[44], CTLA-4 can reduce CD28-mediated PI3K activation, further enhancing the negative signaling through PD-1. Since PD-L1 also interacts with B7-1[45], both αPD-L1 and αCTLA-4 can increase the accessibility of B7-1 to CD28. In our study, HCV-specific CD8 T cells co-expressing PD-1 and CTLA-4 (e.g. from HCV-infected liver) were deeply exhausted and resistant to PD-1 blockade alone, whereas combined PD-1/CTLA-4 blockade had a synergistic effect in restoring their function. However, the two negative regulators may act redundantly to inhibit T cell function in this setting, such that both must be blocked to regain normal functions. The PD-1^+^CTLA-4^+^ phenotype with a functional response to PD-1/CTLA-4 blockade also occurred in circulating HCV-specific CD8 T cells during acute hepatitis C. By contrast, circulating HCV-specific CD8 T cells from chronic HCV patients (e.g. with intermediate PD-1 expression without CTLA-4 co-expression) were functionally augmented by PD-1 blockade alone. These findings suggest that the CTLA-4 and PD-1 pathways are induced early in HCV infection to co-regulate HCV-specific CD8 T cell function in a redundant manner that differs between tissue compartments over the course of infection.

Notably, CD28 was highly expressed in intrahepatic PD-1^+^CTLA-4^+^ CD8 T cells compared to CTLA-4^−^ CD8 T cells. CD28 expression may be induced to counter the inhibitory signals mediated by PD-1 and CTLA-4. Conversely, CTLA-4 may be induced in CD28^+^PD-1^+^CD8 T cells to downregulate the active inflammation at the site of viral replication. In either case, increased CD28 expression may enable greater functional enhancement upon PD-1/CTLA-4 blockade due to unhampered positive signaling through CD28. Indeed, the functional response to PD-1/CTLA-4 blockade was abolished in HCV-specific CD8 T cells by CD28-depletion in our study. Along these lines, direct CD28 costimulation enhanced HCV-specific CD8 T cell IFN-γ response in HCV-monoinfected patients but not in HIV/HCV-coinfected patients with reduced CD28 expression on CD8 T cells in one study[46]. Since loss of CD28 expression is a marker of T cell senescence and functionality[47], detection of CD28 expression in PD-1^+^CTLA-4^+^ CD8 T cells provides an additional marker for reversible functional exhaustion. Collectively, these costimulatory receptors may define a dynamic and complex functional hierarchy for antigen-specific CD8 T cells at various stages and types of viral infections that may respond to distinct therapeutic modulation. If this is correct, combined blockade could have potential therapeutic implications in chronic viral infection, provided it does not trigger autoimmunity.

There are distinct differences between our study and those in human HIV or murine LCMV infections[4],[34]. In peripheral blood of HIV-infected patients, increased CTLA-4 expression with a functional response to CTLA-4 blockade was limited to HIV-specific CD4 but not CD8 T cells. This difference may be explained by the reduced CTLA-4 and CD28 expression in HIV-specific CD8 T cells[34], since the functional response to combined PD-1/CTLA-4 blockade depended on both CTLA-4 and CD28 expression in our study. Alternatively, HIV-specific CD8 T cells might exhibit compartmental differences (e.g. between blood and tissue compartments) similar to HCV. In LCMV-infected mice, LCMV-specific CD8 T cells displayed increased CTLA-4 and PD-1 expression, but responded only to PD-1 but not CTLA-4 blockade *in vivo*. Furthermore, HIV-specific and LCMV-specific T cells were functionally augmented by αPD-L1, without a synergistic response to combined αPD-L1/αCTLA-4[4],[34]. Thus, PD-1 may play a more universal role in antiviral T cell exhaustion whereas the effect of CTLA-4 may differ between viral infections, T cell subsets and even anatomical locations.

In conclusion, both CTLA-4 and PD-1 contributes to HCV-specific T cell exhaustion in a redundant manner in human HCV infection, particularly in HCV-infected liver; this intrahepatic virus-specific T cell dysfunction can be synergistically reversed by combined PD-1/CTLA-4 blockade *in vitro* in a CD4-independent and CD28-dependent manner. These findings provide new insights to the mechanisms that regulate virus-specific T cell dysfunction and suggest that immune exhaustion at the site of antigen expression may be reversed by combined inhibitory receptor blockade.

## Materials and Methods

### Study subjects

All subjects were recruited with informed consent approved by the Institutional Review Boards. All investigations have been conducted according to the principles expressed in the Declaration of Helsinki. Patients were recruited at the Philadelphia Veterans Affairs Medical Center (PVAMC) and the Hospital of the University of Pennsylvania. A total of 47 patients with chronic hepatitis C without HIV coinfection (**group C**) were examined, including 33 cirrhotic patients undergoing liver transplantation and 14 patients with chronic stable HCV infection. Control groups included 10 healthy HCV-seronegative subjects (**group N**), 4 HCV-seropositive but RNA-negative patients with spontaneous resolution of HCV infection without prior antiviral therapy (**group R**) and 6 patients with acute hepatitis C (**group A**) diagnosed by acute serum alanine amino-transferase (sALT) elevation with documented HCV-seroconversion and/or viremic fluctuations greater than 10-fold without prior liver disease as described previously[48]. The patient characteristics are shown in **Table 1**.

**Table 1:** Patient groups.

	Acute (A)	Chronic (C)		Recovered (R)	HCV-negative Controls (N)
		Stable	Transplanted		
	n = 6	n = 14	n = 33	n = 4	n = 10
Sex (M/F)	6/0	14/0	29/4	4/0	6/4
HLA-A2+	4	7	15	3	3
Genotype 1	5	13	32	(-)	(-)
Age (years)*	37	55	54	53	48
HCV RNA (IU/ml)*	4,995,000	940,500	530,000	0	0
ALT (IU/ml)*	1234	40	71	24	28
Albumin (g/dl)*	4.1	4.2	2.4	4.6	4.2
Bilirubin (mg/dl)*	6.3	0.7	2.8	0.8	0.5
Platelets (×10^3^/mm^3^)*	166	237	72	240	264

*: Median values.

### Fluorescent antibodies and reagents

All fluorescent monoclonal antibodies (mAbs) were purchased from BD Bioscience (San Jose, CA) except for: (i) αFoxP3 and αCD28 from eBioscience (San Diego, CA); (ii) FITC-labeled αPD-1 (αCD279; clone EH12.2H7) from BioLegend (San Diego, CA); and, (iii) PE-labeled αPD-1 from the Dana Farber Cancer Institute (Boston, MA). Of note, PD-1 and CTLA-4 expression in all subjects was examined using FITC-labeled αPD-1 (clone M1H4, BD) and PE-labeled αCTLA-4 (αCD152; clone BNI3, BD). In selected subjects, the patterns of PD-1 (low/intermediate/high) and CTLA-4 expression in CD8 T cells were compared using FITC-labeled αPD-1 from BioLegend or PE-labeled αPD-1 from the Dana Farber Caner Institute combined with APC-labeled αCTLA-4 (BD). Dead cells were excluded with 7-AAD. For functional blockade, αPD-L1 mAb (clone 29E.2A3.C6) from the Dana Farber Cancer Institute[13],[49] and αCTLA-4 mAb (clone BNI3; BD)[34] were used.

### Peptides and HLA class I tetramers

The HCV-specific T cell response was measured using a pool of 105 overlapping 15mer peptides spanning the entire NS3 protein derived from HCV genotype 1a[13],[39],[48],[50]. Similarly, the T cell response to influenza virus was examined using 49 overlapping 15mer peptides spanning the conserved matrix M1 protein (residues 1–252) based on the human A/PR/8/34 (H1N1) virus[51]. For HLA-A2+ subjects, the following peptides corresponding to optimal CD8 epitopes were synthesized for antigenic stimulation and tetramer synthesis as described previously [13]: (i) HCV NS3 1073 (CINGVCWTV), NS3 1406 (KLVALGINAV) and NS5B 2594 (ALYDVVSKL); (ii) influenza matrix (GILGFVFTL); (iii) EBV BMLF1 (GLCTLVAML); and, (iv) CMV pp65 (NLVPMVATV).

### Immunophenotyping by flow cytometry

Cells were stained with fluorescent antibodies according to the manufacturer's instructions; events were acquired with a FACSCalibur or FACSCanto (Becton Dickinson, San Jose, CA) and analyzed with FlowJo software (Tree Star Inc., San Carlos, CA). Compensations were established using single color controls. As CTLA-4 is more readily detected in the cytoplasm due to rapid internalization[19],[34],[52], CTLA-4 expression was assessed by intracellular staining following permeabilization[34]. Cutoffs for CTLA-4 expression was defined by isotype control where 99.9% of the events were negative. **Figure S2** further confirms the preferential CTLA-4 expression on PD-1high CD8 T cells with corresponding isotype and unstained controls.

### Isolation of peripheral blood lymphocytes (PBL) and liver-infiltrating lymphocytes (LIL)

PBL were isolated by standard Ficoll-Histopaque (Sigma Chemical Co., St Louis, MO) density centrifugation[39],[53]. LIL were isolated from 20–50 gm of fresh liver explant tissue that was transported in complete media and processed within 24 hours of explant (usually 1–3 hours) as described previously[13]; briefly, this procedure incorporated careful dicing of liver into 5 mm^3^ pieces, incubation of the liver slurry at 37°C with 1 mg/ml collagenase (Type 1a; Roche Molecular) and 1 µg/ml DNase (Sigma Aldrich) for 30 minutes, further mechanical dissociation using the Seward Stomacher 400 Lab Blender (Brinkman Instruments, Westbury, NY), filtration through a 70 µm nylon filter and Ficoll-Histopaque density centrifugation. Control experiments showed that collagenase digestion for 30 minutes did not alter PD-1, CTLA-4, or CD28 expression (data not shown).

### Analysis of antigen-specific T cell expansion and effector function in the presence or absence of PD-1 and/or CTLA-4 blockade

PBL and LIL (2×10^6^ cells/ml/well) were stimulated on day 0 with overlapping HCV NS3 or influenza matrix 15mer peptides (2 µM) in complete media in the presence of isotype control antibodies, αPD-L1[13], αCTLA-4[34] or both αPD-L1 and αCTLA-4 (10 µg/ml for each mAb). Cell cultures were stimulated with rIL-2 (100 IU/ml) on day 4 and examined by flow cytometry on day 7 with CD107a, intracellular cytokine or perforin staining as previously described[13],[54]. For intracellular cytokine staining, expanded PBL and LIL cultures were stimulated for 6 hours with HCV or Flu peptides in the presence of brefeldin A (10 µg/ml) before surface staining, permeabilization and intracellular staining with αIFN-γ and αTNF-α. Antigen-specific CD107a mobilization was quantified by adding FITC-labeled αCD107a before peptide stimulation. In selected HLA-A2^+^ subjects with available cells, PBL and LIL were stimulated with HLA-A2 restricted antigenic peptides (10 µg/ml) with the blocking conditions described above.

In select experiments, antigen-specific IFN-γ^+^ T cell response was quantified by IFN-γ ELISPOT assay in which cultured lymphocytes were stimulated for 45 additional hours with antigenic peptides or control media (200,000 cells/well in triplicates) as previously described[39],[50],[53]. HCV-specific IFN-γ^+^ T cell frequency was calculated by subtracting the mean IFN-γ spot forming units (SFU) in control wells from the mean SFU in antigen-stimulated wells and expressed as IFN-γ SFU/10^6^ cells.

### CFSE proliferation assay

Lymphocytes were labeled with 5 mM CFSE (Molecular Probes, Eugene, OR) as described previously[13],[17] before 7 days of culture with antigenic peptides (10 µg/ml) in the presence of isotype control or blocking antibodies as described above. Cell cultures were stimulated with rIL-2 (100 IU/ml) on day 4 and examined by flow cytometry on day 7 for antigen-specific T cell expansion.

### Depletion of CD4 and CD28

CD4 T cells were depleted from PBL and/or LIL using CD4 Dynabeads (Invitrogen, Oslo, Norway) as previously described[39]. CD28^+^ T cells were depleted by sequentially staining with αCD28-PE (clone CD28.2, BD Pharmingen) and anti-PE Microbeads before separation by AutoMACS (Miltenyi Biotec Inc) as previously described [17]. The efficiency of CD4 and CD28 depletion was >97% (data not shown).

### Statistics

Clinical and immunological parameters were compared using the Mann-Whitney U-test, the paired t-test and the Kruskal-Wallis test. Frequency differences were compared by Fisher's Exact test or the Chi-square test as appropriate. Correlations were tested for significance by the Spearman rank correlation test. P values below 0.05 were considered significant.

## Supporting Information

Figure S1 CTLA-4 and PD-1 expression in intrahepatic CD8 T cells from HCV-seronegative subjects. CTLA-4 and PD-1 expression in gated CD8 T cells from liver explants are shown for 2 HCV seronegative subjects (NC091, NC109) with nonalcoholic steatohepatitis (NASH) and alcoholic cirrhosis and 2 representative chronic HCV patients (C97, C08). Bottom plots show the isotype control staining of the intrahepatic lymphocytes.
(1.26 MB EPS)

Figure S2 Preferential CTLA-4 expression in PD-1high CD8 T cells isolated from blood of an acute hepatitis C patient (A46). The sample on the far right was stained with αCTLA-4, aPD-1, αCD8 and 7-AAD. Two samples on the left were stained with the same antibody cocktail minus anti-CTLA-4 (unstained) or with the addition of the corresponding isotype antibody.
(0.78 MB EPS)

Figure S3 Increased CTLA-4 expression in CD4 T cells is increased in HCV-infected liver. (A) %CTLA-4^+^ expression in CD4 T cells from peripheral blood and liver of 15 chronic (C) patients and blood of 4 HCV-seronegative controls (N). Median %CTLA-4^+^ in CD4 T cells (red horizontal lines): C-blood 6.9% vs. C-liver 17.9% (p<0.0001 by the Mann-Whitney U-test); N-blood 5.6%. Of note, examination of intrahepatic CD4 T cells from 3 HCV seronegative but cirrhotic patients showed similar level of CTLA-4 expression (10.4%, 4.8%, 1.4%) as those in normal control PBL. (B) %FoxP3^+^ in CD4 T cells from blood and liver of 30 chronic (C) HCV patients. Median %FoxP3^+^ in CD4 T cells (red horizontal lines): C-blood 7.6% vs. C-liver 6.3% (p = 0.209 by the Mann-Whitney U-test). (C) Representative FoxP3 expression in CD4 and CD8 T cells from blood and liver of a chronic HCV patient (C97). (D) %FoxP3^−^CTLA-4^+^ CD4 T cells in the liver and blood in chronic HCV patients (blood, unfilled bars; liver, solid bars).
(1.12 MB EPS)
